# Poly(ADP-ribosyl)ated PXR is a critical regulator of acetaminophen-induced hepatotoxicity

**DOI:** 10.1038/s41419-018-0875-4

**Published:** 2018-07-26

**Authors:** Cheng Wang, Wenjing Xu, Yanqing Zhang, Dan Huang, Kai Huang

**Affiliations:** 1 Clinic Center of Human Gene Research, Union Hospital, Tongji Medical College, Huazhong University of Science and Technology, Wuhan, China; 2 Department of Cardiology, Union Hospital, Tongji Medical College, Huazhong University of Science and Technology, Wuhan, China

## Abstract

Acetaminophen (APAP) overdose is the most frequent cause of acute liver failure and remains a critical problem in medicine. PARP1-dependent poly(ADPribosyl)ation is a key mediator of cellular stress responses and functions in multiple physiological and pathological processes. However, whether it is involved in the process of APAP metabolism remains elusive. In this study, we find that PARP1 is activated in mouse livers after APAP overdose. Pharmacological or genetic manipulations of PARP1 are sufficient to suppress the APAP-induced hepatic toxicity and injury, as well as reduced APAP metabolism. Mechanistically, we identify pregnane X receptor (PXR) as a substrate of PARP1-mediated poly(ADP-ribosyl)ation. The poly(ADP-ribosyl)ation of PXR in ligand-binding domain activates PXR competitively and solidly, facilitates its recruitment to target gene *CYP3A11* promoter, and promotes *CYP3A11* gene transcription, thus resulting in increases of APAP pro-toxic metabolism. Additionally, PXR silence antagonizes the effects of PARP1 on APAP-induced hepatotoxicity. These results identifies poly(ADP-ribosyl)ation of PXR by PARP1 as a key step in APAP-induced liver injury. We propose that inhibition of PARP1-dependent poly(ADP-ribosyl)ation might represent a novel approach for the treatment of drug-induced hepatotoxicity.

## Introduction

Acetaminophen (APAP) is one of the most widely used over-the-counter analgesic to relieve mild-to-moderate pain and reduce fever. APAP is generally safe at therapeutic doses; however, an overdose of APAP has led to severe liver failure and intentional or accidental death in many countries^1–3^. A significant number of early studies showed that APAP toxicity stems from its cytochrome P450-dependent metabolism to N-acetyl-*p*-benzoquinoneimine (NAPQI). Normally, hepatic glutathione (GSH) induces the formation of a safely APAP-protein adduct^4,5^. However, toxic doses of APAP lead to GSH depletion and NAPQI accumulation forms covalent bonds with cysteine groups on hepatocytes, which is able to exert harmful effects on liver function^6,7^. CYP1A2, CYP2E1 and CYP3A11 are the most active P450s that convert APAP to NAPQI^8,9^. Evidence supports that the treatment with these P450 enzymes inducers could increase APAP hepatotoxicity and lethality^10,11^. Despite considerable studies of APAP hepatotoxicity mechanisms, pathophysiological response to hepatic injury caused by APAP overdose and ideal approaches are still poorly understood.

Poly (ADP-ribose) polymerase 1 (PARP)-1 is the most abundant isoform of these PARP enzyme family^12^, and accounting for about 90% of total cellular PARP activity^13^. In the nucleus, PARP1 is responsible for post-translational poly(ADP-ribosyl)ation modification that covalently transfers mono- or oligomeric ADP-ribose moieties from NAD^+^ to itself and other acceptor proteins. PARP1 and PARylation have received considerable attention in the recent literature^14,15^. PARP1 acts as a critical indicator, transducer and effector within the DNA damage and responses with a wide spectrum of functions on chromatin remodeling, transcription, energy metabolism, and regulation of cell death^16,17^. Our previous work has shown that PARP1 plays a pivotal role in the pathogenesis of oxidative stress-induced liver diseases, like non-alcoholic fatty liver, CCL4− and bile duct ligation-induced hepatic inflammation and fibrosis, and chronic alcoholic liver diseases^18–21^. However, it remains unclear whether PARP1 is involved in APAP-related liver drug metabolism.

In the present study, we demonstrate that PARP1-mediated poly(ADPribosyl)ation acts as an important regulator in the APAP-induced liver toxicity. And the benefits of PARP1 deficiency in preventing APAP toxicity may be attributed to the regulation of a pattern of metabolic related genes under PXR, which favors a decreased exposure of the host to the toxic APAP metabolites.

## Results

### Poly(ADP-ribosyl)ation accumulation during development of APAP-induced liver toxicity

**Fig. 1:**
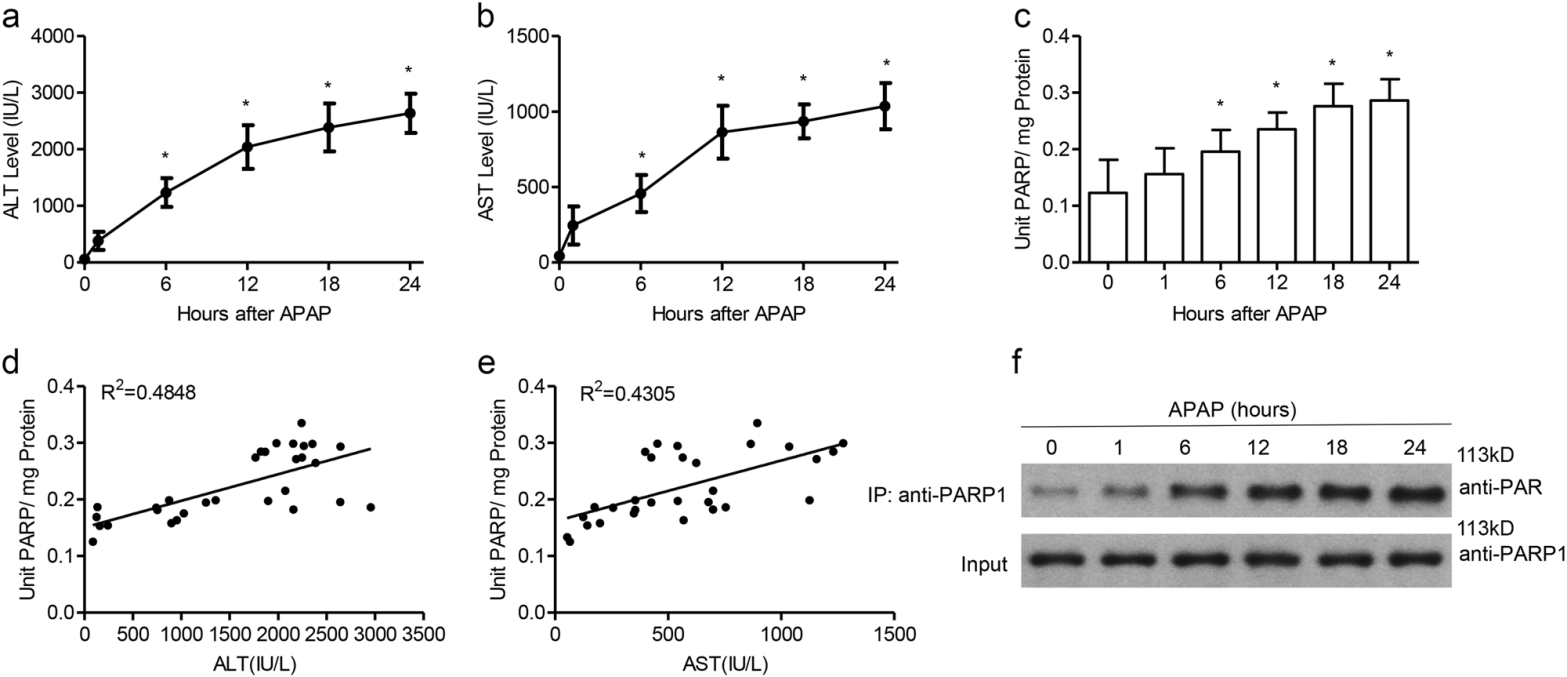
PARP1-dependent poly(ADP-ribosyl)ation is activated in APAP-induced liver toxicity. C57BL/6J mice were injected intraperitoneally with 300 mg/kg APAP or with saline vehicle. Plasma ALT (**a**) and AST (**b**) content were determined at the indicated time. **c** Quantification of PARP activity in the livers of APAP-treated mice at 1, 6, 12, 18, and 24 h. **d**, **e** PARP1 activity and ALT/AST content were positively correlated in APAP-treated mice. *R*^2^ = 0.4848, *P* < 0.01 for ALT; *R*^2^ = 0.4305, *P* < 0.01 for AST. **f** Representative western blot analysis of autopoly(ADP-ribosyl)ated PARP1 in the liver of APAP-treated mice at 1, 6, 12, 18, and 24 h. *N* = 10–12 for each group. *P < 0.01 vs. control

The degree of liver injury after an APAP overdose of 300 mg/kg administration was assessed using serum alanine aminotransferase (ALT) and aspartate aminotransferase ALT over a 0- to 24-h time course, which were markedly increased compared to controls in a time-dependent manner (Fig. 1a, b). Then we assessed the activation of poly(ADP-ribosyl)ation in liver tissues following APAP treatment. Hepatic accumulation of poly(ADP-ribosyl)ation in APAP-treated mice began at 1 h and continued through 24 h (Fig. 1c). We also quantified PARP activity and plasma ALT/AST extent. As shown in Fig. 1d, e, a positive correlation between PARP activity and the extent of plasma ALT/AST content was observed (*R*^2^ = 0.4848, *P* < 0.01 for ALT; *R*^2^ = 0.4305, *P* < 0.01 for AST), suggesting that PARP activation was strongly associated with the severity of liver injury. The preferred and first target of PARP1 enzymatic activity is PARP-1 itself^13^, so we checked the auto-modification level, which reflects cellular PARP1 activity, in APAP-treated liver tissues. Immunoprecipitation (IP) assay results showed that the auto-poly(ADP-ribosyl)ation level of PARP1 was dramatically increased along with time (Fig. 1f). Taken together, PARP1-dependent poly(ADP-ribosyl)ation is activated in APAP-induced liver injury.

### PARP inhibitor ameliorates liver injury and toxic APAP metabolites

**Fig. 2:**
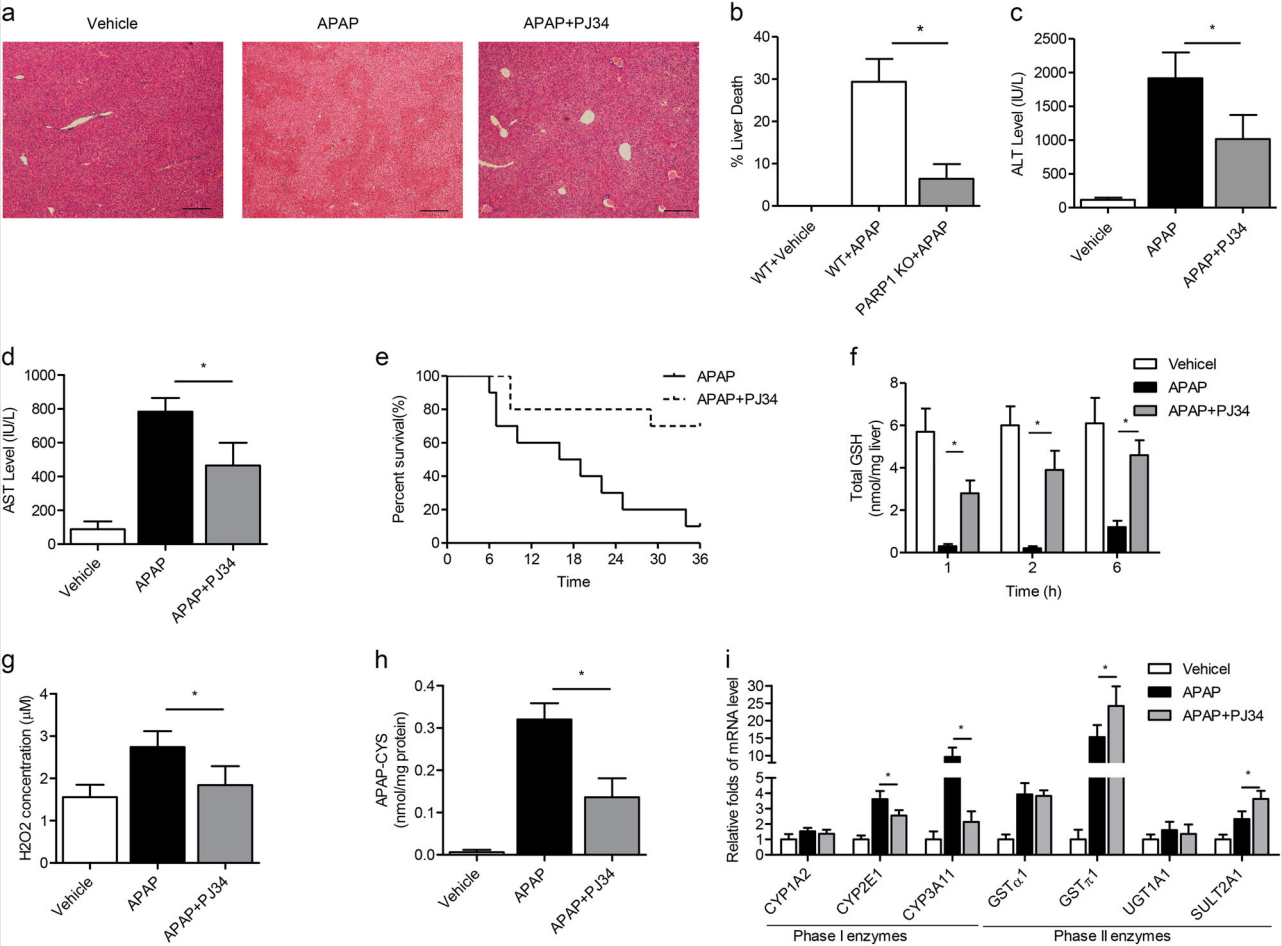
PARP inhibitor PJ34 attenuates APAP metabolism and liver toxicity. C57BL/6J mice were injected intraperitoneally with PJ34, and then administered with 300 mg/kg APAP injection. Blood and tissue were collected at indicated hours after APAP injection. **a** Representative H&E staining of liver sections from Vehicle, APAP and APAP plus PJ34-treated mice. **b** Quantification of liver necrosis area. Serum levels of (**c**) ALT and (**d**) AST activity. **e** Mice were treated with a lethal dose of APAP (1 g/kg). Survival was followed for 36 h post-administration. **f** Total hepatic GSH levels at indicated time points after APAP treatment. **g** Total hepatic H2O2 concentrations and **h** APAP-cysteine levels at 6 h after APAP treatment. **i** The hepatic expressions of Phase I (*CYP1A2*, *CYP2E1* and *CYP3A11*) and Phase II enzyme genes (*GSTα1*, *GSTπ1*, *UGT1A1* and *SULT2A1*) were measured by real-time PCR assays. *N* = 10–12 for each group. *P < 0.01 vs. APAP

To purpose the role of poly(ADPribosyl)ation in APAP-induced liver injury, we first utilized PARP inhibitor PJ34 (9N-(6-oxo-5,6-dihydrophenanthridin-2-yl)-N,N-dimethylacetamide) on APAP mice. Male C57BL/6 J mice were injected with PJ34 just before APAP administration. Results showed that PJ34 could rescue mice from APAP-induced liver injury, as revealed by decreased plasma levels of ALT and AST. Hepatic necrosis was also relieved by H&E staining and the necrotic area was substantially smaller compared to mice given only APAP (Fig. 2a–d). In addition, we used 1 g/kg APAP to challenge the mice to detect the survival rate upon PJ34 treatment. PJ34 showed less susceptibility to APAP injury as manifested by improved survival than that in only APAP that killed 80% WT animals within 24 h (Fig. 2e). Hepatic GSH deletion is a hallmark of excessive NAPQI generation during APAP-induced liver toxicity. We discovered that PJ34 drastically recovered the extent of hepatic GSH depletion due to APAP at early time points (1, 2 and 6 h) (Fig. 2f). Since GSH regulates redox-sensitive components of signal transduction cascades and loss of GSH destroys redox balance^22^, H2O2 levels from liver were then detected at 6 h after administration of APAP. Our results showed that APAP treatment alone significantly raised hepatic H_2_O_2_ levels compared with that in vehicle group, while PJ34 supplement suppressed the increased H_2_O_2_ level in APAP-treated mice (Fig. 2g). Consistently, we found that the hepatic concentrations of APAP-cysteine, APAP metabolites that indicate the formation of toxic metabolite, were all decreased in PJ34 mice compared with APAP-treated mice (Fig. 2h), all suggesting that PARP might affect the formation and toxicity of NAPQI in APAP-induced liver injury.

To explore whether PAPR1 inhibition interfered with APAP metabolism and toxicity, we measured the mRNA and protein levels of major APAP-metabolizing enzymes in vehicle-, APAP- and APAP + PJ34-treated mice. Phase I enzymes has been known to facilitate the formation of toxic APAP metabolites. As shown in Fig. 2i and Supplementary Fig.1a, b, the expressions of CYP2E1 and CYP3A11 were all reduced, whereas CYP1A2 expression remained largely unchanged under PJ34 treatment. Among anti-toxic Phase II enzymes, the expression of GSTα1, GSTπ1 and SULT2A1 were increased after APAP treatment, but PJ34 further enhanced the upregulation of GSTπ1 and SULT2A1 expression (Fig. 2i and Supplementary Fig.1a, b). Furthermore, the increased enzyme activities of CYP2E1 and CYP3A11 were also significantly suppressed by PJ34, accompanied with their expressions (Supplementary Fig.1c, d). Collectively, these results suggest that the protective effects against APAP by PARP inhibition are mainly attributed to altering APAP metabolism.

### PARP1-deficiency mice are rescued from toxic APAP metabolites and liver injury

**Fig. 3:**
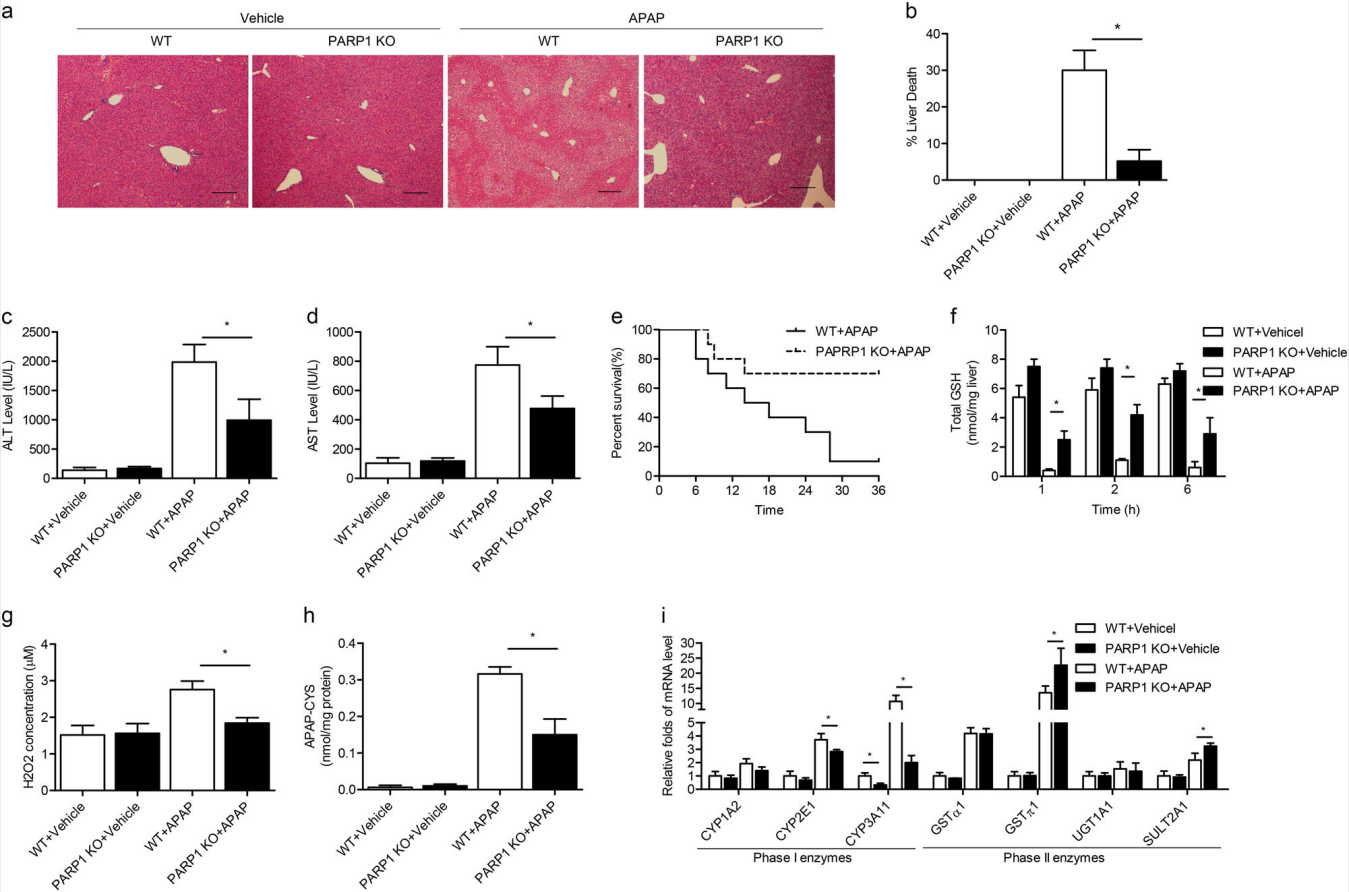
PARP1^−/−^ mice confers resistance to APAP-induced hepatotoxicity. WT and PARP1^−/−^ mice were injected intraperitoneally with APAP (300 mg/kg). Blood and tissue samples were collected. **a** Representative H&E staining of liver sections from WT and PARP1^−/−^ mice. **b** Quantification of liver death area. Serum levels of (**c**) ALT and (**d**) AST activity. **e** Mice were treated with a lethal dose of APAP (1 g/kg). Survival was followed for 36 h post-administration. **f** Total hepatic GSH levels at indicated time points after APAP treatment. **g** Total hepatic H2O2 concentrations and (**h**) APAP-cysteine levels at 6 h after APAP treatment. **i** The hepatic expression of Phase I (*CYP1A2*, *CYP2E1* and *CYP3A11*) and Phase II enzyme genes (*GSTα1*, *GSTπ1*, *UGT1A1* and *SULT2A1*) was measured by real-time PCR assays in WT and PARP1^−/−^ mice. *N* = 10–12 for each group. *P < 0.01 vs. APAP

The hepatic protective effects of pharmacological inactivation of PARP activity prompted us to determine whether PARP1 deficiency mice would have a similar effect in preventing APAP toxicity. Similarly, PARP1^−/−^ mice showed less histological liver necrosis and lower serum levels of AST and ALT compared to WT mice in APAP-treated counterparts (Fig. 3a–d). And the survival rates of PARP1^−/−^ mice after APAP stimulation were nearly 70%, confirming that PARP1 deficiency significantly improved survival after APAP administration (Fig. 3e). As for APAP metabolism, baseline GSH levels were similar between WT and PARP1^−^/^−^ mice. Under APAP treatment, the rate and extent of GSH depletion and re-synthesis were highly improved in PARP1^−/−^ mice (Fig. 3f), as well as reduced hepatic H2O2 levels (Fig. 3g). Meanwhile, we also found that PARP1 deficiency could decrease the hepatic concentrations of APAP-cysteine (Fig. 3h). Finally, we measured phase I and phase II enzymes known to facilitate the formation of toxic APAP metabolites. Consistent with PJ34, upon APAP treatment, PARP1^−^^/−^ mice showed lower expressions of CYP2E1 and CYP3A11, along with decreased enzyme activities, whereas the expression of the CYP1A2 nearly unchanged. And GSTπ1 and SULT2A1 levels were increased in PARP1^−^^/−^ mice (Fig. 3i, Supplementary Fig.2). These results strongly suggest that PARP1 deficiency might halt liver injury via regulation of APAP metabolism.

### PARP1 binds to PXR

**Fig. 4:**
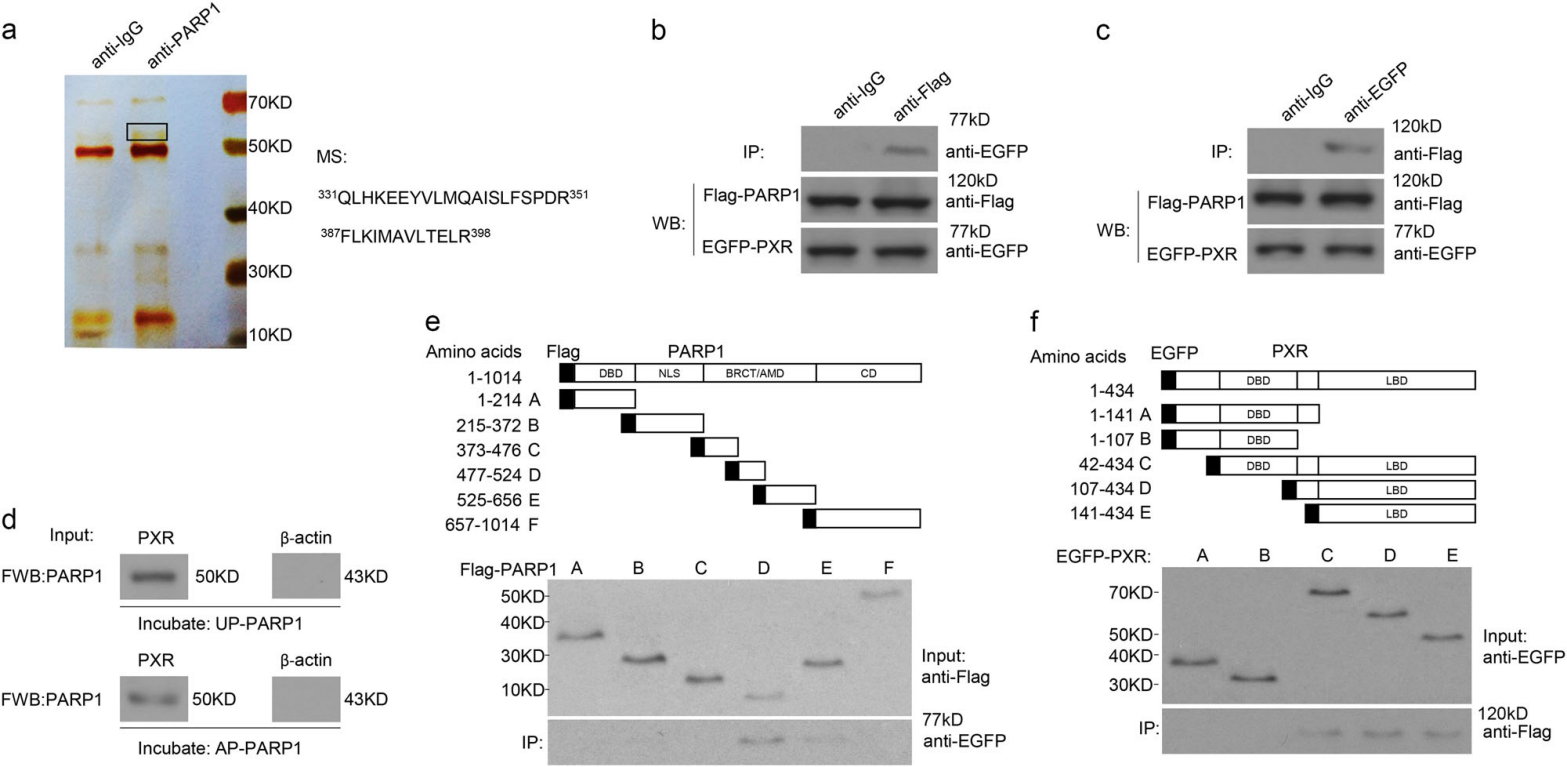
PARP1 binds directly to PXR. **a** Immunoaffinity purification of PARP1-containing protein complexes from the liver of APAP-treated mice were subjected to mass spectrometry analysis. Black letters indicate the peptides identified. **b**, **c** HepG2 cells were transfected with EGFP-tagged PXR and Flag-tagged PARP1. **b** Coimmunoprecipitation assays of Flag-tagged PARP1-bound proteins, followed by western blot assays using an anti-EGFP antibody. Nonspecific IgG served as a negative control. **c** Coimmunoprecipitation assays of EGFP-tagged PXR-bound proteins, followed by western blot assays using an anti-Flag antibody. Nonspecific IgG served as a negative control. **d** Far-western blot assays of recombinant PXR protein. UP-PARP1 or AP-PARP1 was used as a probe. β-Actin protein served as a negative control. **e** Diagram of Flag-tagged human PARP1 with its domains: DNA-binding domain (DBD), nuclear localization signal (NLS), BRCA1 C terminus (BRCT)/automodification domain (AMD), and catalytic domain (CD). Fragments A–F with their amino acid coordinates are listed. HepG2 cells were transfected with EGFP-tagged full-length PXR and Flag-tagged PARP1 mutants. Coimmunoprecipitation assays demonstrated the specific binding of PXR to the BRCT/AMD of PARP1. **f** Diagram of EGFP-tagged human PXR with its domains. LBD, ligand-binding domain. Fragments A to E with their amino acid coordinates are listed. HepG2 cells were transfected with Flag-tagged full-length PARP1 and EGFP-tagged PXR mutants. Coimmunoprecipitation assays demonstrated the specific binding of PARP1 to the LBD of PXR

To clarify the precise mechanism how PARP1 regulates APAP metabolism, we explored PARP1-interacting proteins in the nuclear extracts from APAP-treated liver. After immunoprecipitation with anti-PARP1 antibody, silver staining assay showed that there existed an additional band with molecular weight around 50 kDa in eluted proteins between anti-PARP1 vs. anti-IgG groups. The corresponding protein band was thereafter subjected to LC-MS/MS assay. After elimination of false-positives presenting in controls, this protein was identified as PXR, a key regulator of the detoxification of xeno- and endobiotics^23^ (Fig. 4a). To further confirm the association of PARP1 and PXR in the nuclear extracts, we performed co-immunoprecipitation (co-IP) assay. Nuclear extracts from HepG2 cells co-transfected with Flag-PARP1 and EGFP-PXR were subjected to co-IP with an antibody specific for Flag or EGFP. Western blot assays showed that PXR was co-precipitated with PARP1, and vice versa (Fig. 4b, c). Next, we investigated whether unpoly(ADP-ribosyl)ated PARP1 (UP-PARP1) or autopoly(ADP-ribosyl)ated PARP1 (AP-PARP1) could bind specifically to PXR. In a cell-free system, far-Western blot assay showed that recombinant PXR protein (50 kDa) could bind directly to either UP-PARP1 or AP-PARP1 (Fig. 4d). Furthermore, we explored the functional domains mediating the PARP1–PXR interaction. A series of PARP1 deletion mutants were made. We mapped exogenous PXR exclusively to the BRCA1 C terminus (BRCT)/automodification domain (AMD) of PARP1 (Fig. 4e). In contrast, using PXR deletion mutants, we observed that PARP1 directly interacted with the C-terminal ligand-binding domain (LBD) of PXR (Fig. 4f). Thus, we conclude that PXR directly binds to PARP1 via the LBD and the central BRCT/AMD.

### PXR can be poly(ADP-ribosyl)ated by PARP1

**Fig. 5:**
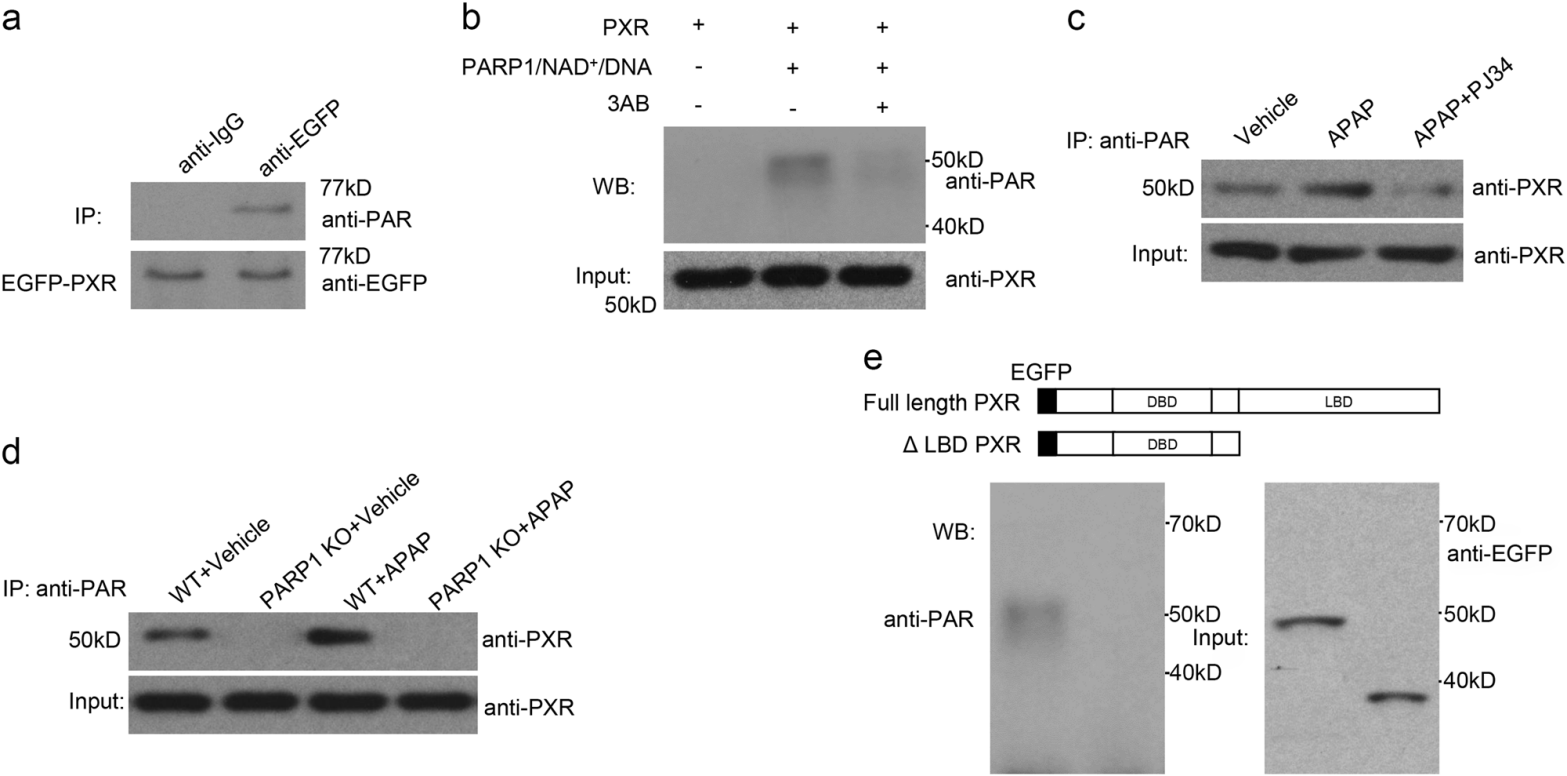
PXR can be poly(ADP-ribosyl)ated by PARP1. **a** HepG2 cells were transfected with EGFP-tagged PXR. Nuclear extracts from HepG2 cells were subjected to an immunoprecipitation assay with an anti-PXR antibody, followed by a western blot assay using an anti-PAR antibody. **b** Recombinant PXR protein were incubated either with a vehicle, or PARP1, NAD+, and activated DNA, or with PARP1, NAD^+^, activated DNA, and 3AB, as indicated. Western blot assays were used to detect the poly(ADP-ribosyl)ation levels of PXR.(**c**) Liver nuclear extracts from Vehicle, APAP and APAP plus PJ34-treated mice were subjected to an immunoprecipitation assay with an anti-PAR antibody, followed by a Western blot assay using an anti-PXR antibody. **d** Liver nuclear extracts from Vehicle or APAP in WT and PARP1^−^^/−^ mice were subjected to an immunoprecipitation assay with an anti-PAR antibody, followed by a western blot assay using an anti-PXR antibody. **e** Diagram of EGFP-tagged human PXR with or without LBD domain (ΔLBD PXR). Full-length EGFP-PXR or lacking LBD domain EGFP-PXR protein were incubated with recombinant PARP1 protein in the presence of activated DNA and NAD^+^. Poly(ADP-ribosyl)ation of FL PXR or ΔLBD PXR was detected by a western blot assay with an anti-PAR antibody

PARP1 is well-known for catalyzing target proteins poly(ADP-ribosyl)ation^24^. We therefore speculated whether PARP1 could poly(ADP-ribosyl)ate PXR. Nuclear extracts of EGFP-PXR-transfected HepG2 cells were subjected to IP with anti-EGFP antibody. Western blot assays with an specific poly(ADP-ribose) polymer (PAR) antibody revealed that PXR could indeed be poly(ADP-ribosyl)ated (Fig. 5a). More importantly, incubation of recombinant PXR with PARP1, activated DNA and NAD^+^ led to strong poly(ADP-ribosyl)ation of PXR in a cell-free system, which was inhibited by the addition of the PARP1 inhibitor 3AB (Fig. 5b). On account of PARP1 activation after APAP treatment, we then detected the poly(ADP-riboyl)ation of PXR in the livers of APAP-treated mice. Using IP assay as indicated, we demonstrated that APAP versus vehicle treatment displayed a significant increase in PXR poly(ADP-riboyl)ation. When PARP1 was inhibited by PJ34 or knockout the levels of PXR poly(ADP-riboyl)ation were markedly decreased (Fig. 5c, d), all indicating that PXR could be poly(ADP-ribosyl)ated by PARP1 in vitro and in vivo.

Given that the ligand-binding domain mediated the interaction of PXR with PARP1, we sought to determine whether PXR was poly(ADP-ribosyl)ated by PARP1 via this domain. We constructed purified PXR mutant lacking LBD domain (ΔLBD PXR) protein. In a cell-free system, recombinant full length or lacking LBD domain PXR protein were incubated with recombinant PARP1 protein, NAD^+^, and actived DNA, and subjected to western blot assay, which demonstrated that PXR purified protein that lacking LBD domain could not be poly(ADP-ribosyl)ated by PARP1 (Fig. 5e), suggesting that LBD of PXR was a bona fide substrate for PARP1-mediated poly(ADP-ribosyl)ation.

### Poly(ADP-ribosyl)ated PXR promotes *CYP3A11* transcriptional activation

**Fig. 6:**
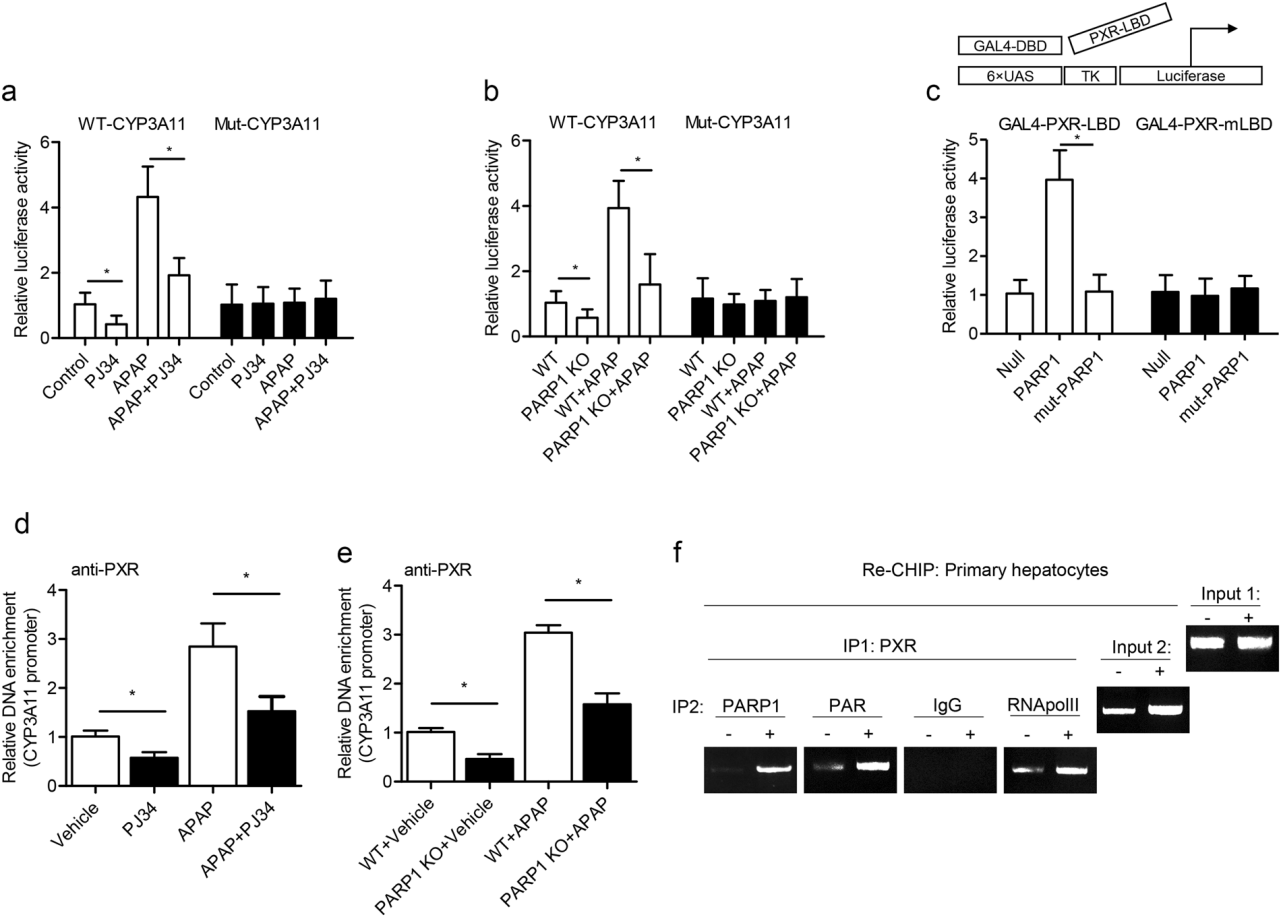
Poly(ADP-ribosyl)ated PXR promotes its activation and recruitment to *CYP3A11* gene. **a** Relative CYP3A11 or mutant CYP3A11 (mut-PXRE) promoter luciferase reporter activity in primary mice hepatocytes treated with PJ34(15 µM) or APAP(300 µM) for 24h. **b** Relative CYP3A11 or mutant CYP3A11 (mut-PXRE) promoter luciferase reporter activity in primary WT or PARP1^−/−^ mice hepatocytes with or without APAP treatment (300µM; 12 h). **c** HepG2 cells were transfected with the GAL4 reporter plasmid together with Gal4-PXR-LBD or Gal4-PXR-LBD mutant, and then exposed to activated PARP1 or inactivated PARP1 (mut-PARP1) for 24 h. Luciferase activity was assayed, and normalized. The data were represented by mean ± SEM of four independent experiments. *P < 0.01 vs. APAP. **d** CHIP-PCR assays using an anti-PXR antibody for amplification of CYP3A11 promoters in primary mice hepatocytes treated as indicated. **e** CHIP-PCR assays using an anti-PXR antibody for amplification of CYP3A11 promoters in primary WT or PARP1^−/−^ mice hepatocytes. **f** In re-CHIP assays, chromatin was first immunoprecipitated with an anti-PXR antibody and was then re-immunoprecipitated with an anti-PAR antibody, an anti-PARP1 antibody, IgG, or an anti-RNA Pol II antibody. IgG served as a negative control

In the nuclear, PXR binds directly to PXRE in the promoters of drug metabolic genes to regulate their transcription and expression^25^. And PXR was reported to induce CYP3A expression^26^. So we went on exploring the functional effects of PARP1-mediated poly(ADP-riboyl)ation on PXR transcriptional responses on CYP3A11. We applied a luciferase reporter assay and found that APAP treatment resulted in increased luciferase activity of the CYP3A11 reporter but not that of the mut-PXRE CYP3A11 luciferase reporter, while treatment with PJ34 suppressed the activated responses of APAP (Fig. 6a). Consistently, PARP1^−/−^ hepatocytes displayed a significant repression on APAP-induced CYP3A11 promoter activity compared with WT hepatocytes (Fig. 6b). PXR is known to be a ligand-activated transcription factor. While the ligand-binding domain of PXR mediated the interaction with PARP1, leading us to suspect whether PARP1-activated PXR in LBD would act like a ligand. Using yeast one-hybrid system, transcription factor GAL4-DBD was co-transfected with GAL4-PXR-LBD or Gal4-PXR-LBD mutant, a mutant deficient in its ligand-dependent activation domain, and the data showed that only activated PARP1 not inactivated PARP1 (mut-PARP1, a catalytically inactive mutant of PARP1) could activate GAL4-PXR-LBD to promote the target luciferase. And the PARP1-induced activity was diminished when present with GAL4-PXR-LBD mutant, all indicating that PARP1 bound to and poly(ADP-ribosyl)ated PXR might competitively alter the affinity between PXR and related PXR ligands to enhance the transactivational activity of PXR (Fig. 6c).

To further investigate how poly(ADP-ribosyl)ation of PXR inhibited its transcriptional function, we studied the effects of PXR poly(ADP-ribosyl)ation on its recruitment to the target promoters. CHIP assays revealed that exposure to APAP led to a significant increase in recruitment of PXR to CYP3A11 promoter in hepatocytes, which was reversed by PJ34 treatment (Fig. 6d). Similarly, after APAP treatment, PARP1^−/−^ hepatocytes displayed a significant reduction of recruitment of PXR to the CYP3A11 promoter compared with that in WT hepatocytes (Fig. 6e), suggesting that PARP1-dependent poly(ADP-ribosyl)ation accelerated the recruitment of PXR to its target promoter. To solid this speculation, re-ChIP assays, in which chromatin was re-precipitated using an anti-PAR or PARP1 antibody, were performed, and showed that both PARP1 and poly(ADP-ribosyl)ated PXR complex could be detected in the CYP3A11 promoter (Fig. 6f). Taking together, we conclude that poly(ADP-ribosyl)ation might act like a ligand to activate PXR and further facilitates the formation of the PXR-promoter complex to promote its transactivation.

### PXR mediates the effects of PARP1 on APAP-induced liver toxicity

**Fig. 7:**
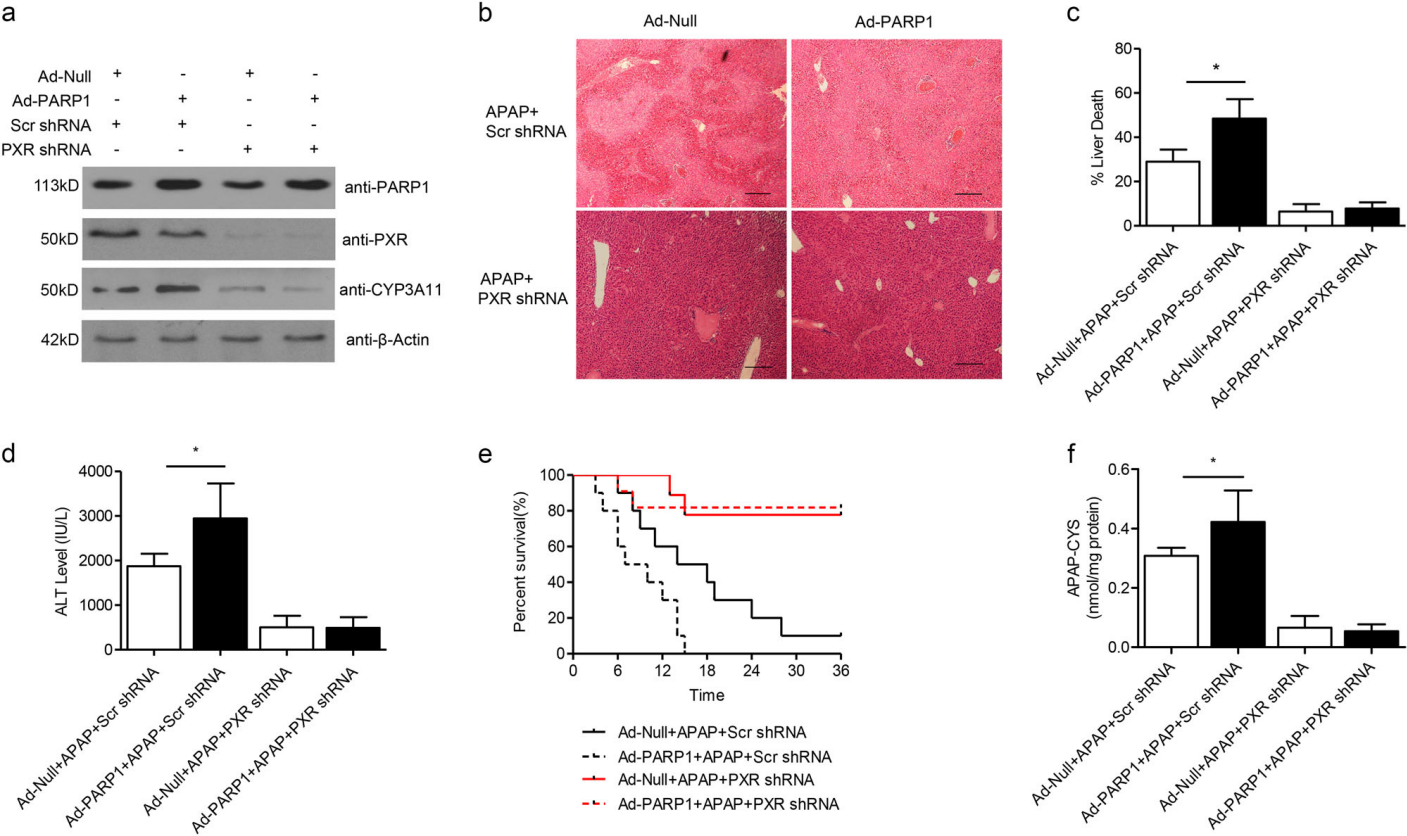
PXR acts as a critical mediator on the effects of PARP1 in APAP-induced liver toxicity. C57BL/6 J mice were pre-delivered Ad-Null or Ad-PARP1, together with Ad-Scr shRNA or Ad-PXR shRNA adenovirus through tail vein, and then exposed to APAP treatment (300 mg/kg). Blood and tissue were collected at indicated times. **a** Representative western blot analysis of hepatic PARP1, PXR and CYP3A11 expressions. **b** Representative H&E staining of liver sections from indicated groups. **c** Quantification of liver death area. **d** Serum levels of ALT activity. (**e**) Mice were treated with a lethal dose of APAP (1 g/kg). Survival was followed for 36 h post administration from indicated groups. **f** Hepatic APAP-cysteine levels at 6 h after APAP treatment from indicated groups. *N* = 10–12 for each group. *P < 0.01 vs. APAP

We finally explored whether PXR/CYP3A11 signaling was implicated in mediating the effects of PARP1 on hepatic APAP metabolism and liver injury. We selectively knocked down PXR expression in mice. As expected, CYP3A11 protein level was decreased as well (Fig. 7a). In comparison with Ad-Null, Ad-PARP1 greatly aggravated liver injury, evident by HE staining for hepatic necrosis and plasma ALT content. However, when PXR was silenced, the above liver injury worsen by PARP1 were effectively reversed (Fig. 7b–d), in agreement with PXR null mice^27–30^. Similarly, excess PARP1 could not facilitate the APAP-induced mice death in PXR-depleted mice (Fig. 7e). Moreover, PXR depletion also dramatically abrogated hepatic APAP-cysteine increase for APAP metabolism (Fig. 7f). In line with these results, CYP3A11 silence consistently disturbed the exaggerated APAP liver injury by PARP1 (Supplementary Fig.3). Cumulatively, these data indicate that PXR-CYP3A11 indeed mediates the effects of PARP1 in APAP-induced liver injury and metabolism.

## Discussion

PARP1 is a ubiquitous multifunctional nuclear enzyme. PARP1-dependent poly(ADP-ribosyl)ation has been reported to be involved in cardiac ischemia/reperfusion injury, atherosclerotic plaque stability, fatty liver prevention and cell homeostasis^19,31–34^. In this study, we found that hepatic PARP1 activity is dramatically increased in APAP-treated mouse liver. Treatment with a PARP inhibitor PJ34 or PARP1 depletion prevented APAP-induced liver toxicity and suppressed APAP metabolism. Mechanically, we discovered that PXR could bind to PAPR1 and was a substrate of PARP1. Poly(ADP-ribosyl)ation of PXR activated its target metabolic gene CYP3A11 expression, promoting toxic APAP metabolites production, and thus worsening APAP-induced liver toxicity. More importantly, PXR or CYP3A11 knockdown could abrogate the deterious effects due to excess PARP1 on APAP liver injury.

It is well established that oxidative/nitrative stress-induced excessive PARP activation in cells leads to energetic collapse and cell death^35^. Liver is a major organ attacked by ROS/RNS. A variety of risk factors, including drugs, alcohol, irradiation, and environmental pollutants, may induce oxidative stress in liver and then result in severe liver diseases^36^. Shiobara et al. showed that dramatically increased PARP1 expression and PAR accumulation in hepatic tissues from patients with liver cirrhosis and hepatoma^37^. Our group and other researchers have demonstrated that PARP is over-activated in livers of subjects with alcoholic and non-alcoholic steatohepatitis. Pharmacological inhibition of PARP1 enzymes attenuates high fat or alcohol-induced liver injury, fat accumulation, inflammation and fibrosis in preclinical models of liver disease^18–21^. In this study, our current results showed that PARP1-dependent poly(ADP-ribosyl)ation was activated in APAP-induced liver injury, and indicated a positive relation with the extent of liver injury. Notably, in agreement with previous reports on liver-related disease, PARP inhibitors or PARP1 deficiency also exerted beneficial role for relieving APAP metabolism and liver injury.

However, earlier work done by Cover et al.^38^ showed that PARP inhibitor 3AB significantly reduced liver injury at 6 h after APAP administration, but neither PARP1^–/–^ mice nor animals treated with the specific PARP-1 inhibitor 5-AIQ were protected against AAP-induced liver injury. Many factors, like mouse background, fasting period, biometric calculation of required cohorts, etc, can influence the experiments^39^. Firstly, PARP1^−/−^ mice in our study were obtained by crossing PARP1^−/−^ mice (129 Sv, from Jackson Laboratory) with C57BL/6 J mice 10 generations to generate PARP1^–/–^ mice in C57BL/6 J background. It is known that different mouse strains from different genetic backgrounds develop significantly less or more injury. As such, C57BL/6 mice show enhanced liver injury and increased expression of tumor necrosis factor-α (TNF-α) compared with BALB/c mice. Secondly, Cover et al. detected the effects of 3AB or PARP1 depletion on liver injury at 6 h, while increased PARP1 activity was observed at 12 and 24 h after APAP, and we found that the protective effects of PJ34 and PARP1 knockout were mainly at later period (24 h) after APAP administration. Thirdly, given the huge variability of APAP-induced liver injury model, a variance in transaminases of around 25% in wild mice 12 h after APAP application required more sample size in each group. We applied nearly 10–12 mice per group compared to 5 animals per group in their study to get more accurate results. In addition, PARP1 is reported to be responsible for 90% of the total cellular PARP activity^24^. PARP1^−/−^ mice exhibit protective effect for APAP-induced liver injury, which also may be due to the compensatory effects of PARP2 over-activation. Further investigations are necessary to explore the differences. Thus, PARP1 depletion possibly protects APAP-induced liver injury.

APAP is one of the most commonly used analgesics and reportedly the most common cause of acute liver failure. It is still surround our understanding of this pathological process. P450 enzymes catalyzed the metabolic activation of acetaminophen, and the reactive metabolite for hepatotoxicity is generally believed to be NAPQI^8,11^. In this study, we observed inhibition of PARP1 using either pharmacological inhibition (PJ34) or PARP1 deficiency could reduce the expression of CYP3A11 and CYP2E1, not CYP2A1 and resulted in increased resistance to APAP toxicity. More importantly, we provided evidence that decreased CYP3A11 was attributed to suppressed PXR transcriptional activity by PARP1 inhibition. The hepatic concentrations of APAP-cysteine, APAP metabolites that indicate the formation of toxic metabolites, were also decreased in PJ34 and PARP1^−/−^ mice. Phase II drug metabolizing enzymes play an important role in serving as a detoxifying step during drug metabolism. Our results showed that PARP1 deficiency mildly upregulated the expressions of two major enzymes, GSTπ1 and SULT2A1. Several lines of evidence have indicated that liver-enriched nuclear receptors including liver X receptor (LXR) or farnesoid X receptor (FXR) could activate Gstπ1 and Sult2a1 transcription though directly binding to these promoters^28,40^. Our group and other groups have reported that PARP1 could interact with and poly(ADP-ribosyl)ated LXR and FXR to mediate their transcriptional activities. We thus concluded that the hepatic protective effect of PARP1 deficiency may result from the suppression of pro-toxic P450s mainly and the induction of anti-toxic Phase II enzymes. But it still remains to be determined whether PARP1 regulates the expression of these Phase II enzymes through LXR- or FXR-dependent post-transcriptional or other mechanism^20,32^.

PARP1 has been reported to form definitive structures through intramolecular interactions and can change the structure of receptor proteins^13^. In our study, we demonstrated that PARP1 could directly bind with PXR and Poly(ADP-ribosyl)ate PXR to alter its function. More interestingly, we discovered that the ligand-binding domain of PXR mediated the binding and poly(ADPribosyl)ation by PARP1. The LBD of PXR has been reported to be critical for specific and constitutive activation. Given that poly(ADP-ribosyl)ated amino acid residues from PXR might change the spatial conformation of the Ligand-binding domain, we conjectured that poly(ADP-ribosyl)ation might competitively promote the activation of PXR just like a ligand. In line with this notion, PARP inhibitor or PARP1 deficiency inhibited the APAP-induced poly(ADP-ribosyl)ation of PXR, and thus antagonized the transactivation of PXR. Overall, in our models, we firstly proved that under APAP-treated condition, activated PARP1 could poly(ADP-ribosyl)ate PXR. Poly(ADPribosyl)ated PXR undergoes a conformational change that unfold the “docking” site in the LBD, recruit the coactivator complexes, and then enhance the PXR signaling pathway to aggravate APAP metabolism.

In summary, the present study demonstrates that PARP1, via activating PXR and promoting pro-toxic P450 enzymes, may represent a potential therapeutic target for the prevention and treatment of APAP-induced liver toxicity.

## Materials and methods

### Animals

PARP1^−/−^ mice (129 Sv) from Jackson lab were crossed with C57BL/6J mice ten generations to generate PARP1^−/−^ in C57BL/6J background. C57BL/6J and PARP1^−/−^ (C57BL/6J background) mice were bred and housed under specific pathogen-free conditions in the animal facility. Sex-matched littermates between 12 and 16 weeks of age were used. Animal experiments were carried out in accordance with the Animal Care Committee of Huazhong University of Science and Technology. Mice were fasted for 12 h prior to APAP injection. APAP (Sigma-Aldrich, Beijing, China), at a dose of 300 mg/kg or 1 g/kg was injected intraperitoneally. In some experiments, mice were injected intraperitoneally with PJ34 (10 mg/kg per day, Selleck, Shanghai, China) 12 h and 0 h before APAP administration. Liver tissues and blood samples were collected at 1, 6, 12, 18, and 24 h after APAP injection.

### Analysis of ALT and AST activity

Plasma samples were diluted and AST/ALT enzyme levels were analyzed at the Department of Clinical Chemistry from Wuhan Union Hospital, Tongji Medical College.

### PARP activity assay

PARP1 activity was assayed using the PARP universal colorimetric assay kit (Trevigen, Maryland, USA), based on the incorporation of biotinylated ADP-ribose into histone proteins. Liver lysates containing 50 μg of protein were loaded into a 96-well plate coated with histones and biotinylated poly(ADP-ribose), allowed to incubate for 1 h, treated with horseradish peroxidase (HRP)-conjugated streptavidin, and read at 450 nm in a spectrophotometer.

### Co-immunoprecipitation

Briefly, 500 µg of protein extracts were incubated with the indicated antibodies against PARP1 (Cell Signaling Technology,9532), Flag (Sigma-Aldrich,F1804), EGFP (Abnova,PAB8931), PXR (Proteintech,15607–1-AP), PAR (Trevigen,4335-MC-100) or unspecific IgG at 4 °C overnight, and protein-A/G agarose (EMD Milipore, IL, USA) was added for another 2 h at 4 °C. The immunoprecipitates were pelleted by centrifugation and washed with lysis buffer (50 mM Tris, 150 mM NaCl, 1% NP-40, 1% sodium deoxycholate) four times. The pellets were then suspended in SDS loading buffer and subjected to western blot assays.

### Western blot assays

Proteins were extracted and measured using the BCA protein assay kit (Thermo, CA, USA). After denaturation and SDS-PAGE electrophoresis, separated proteins were transferred to PVDF membranes and probed with primary antibodies against PAR (Trevigen, 4335-MC-100), PARP1(Trevigen, 4338-MC-50), CYP1A2 (Proteintech, 19936-1-AP), CYP2E1 (Proteintech,19937-1-AP), CYP3A11 (Proteintech,18227-1-AP), GSTα1 (Proteintech,14475-1-AP), GSTπ1 (Proteintech,15902-1-AP), UGT1A1 (Abcam, ab62600), SULT2A1 (Abcam, ab194113), Flag(Cell signaling technology, 2368), EGFP(Sigma, G1544), PXR(Santa Cruz, sc-48340), β-actin (Abcam, ab8226) and a secondary HRP-conjugated IgG antibody. Chemiluminescence signals were detected by the Image Lab software (Bio-Rad, CA, USA).

### H&E staining

Liver specimens were fixed in 4% buffered formalin, and embedded in paraffin (Sakura, Tokyo, Japan). Tissue sections (4μm) were prepared and stained with H&E. Necrotic areas were quantified by area fraction analysis (Image J).

### Assessment of drug metabolism

Total GSH content was measured by GSH-Glo Glutathione Assay (Promega, WI, USA). APAP-cysteine protein adducts were measured as previously described^27^. Briefly, liver tissues were homogenized and dialyzed to remove the free APAP-cysteine, then digested with protease, and the amount of APAP-cysteine was measured using high performance liquid chromatography-tandem mass spectrometry. Hepatic H2O2 levels were accessed using the PeroxiDetect kit (Sigma-Aldrich) according to the manufacturer’s protocol.

### RNA isolation and real-time PCR

Total RNA from liver tissues was isolated using Trizol reagent (Applied Biosystems, CA, USA). cDNA Reverse Transcription Kit (Applied Biosystems) was used to prepare cDNA. Real-time PCR was performed on a StepOnePlus Real-Time PCR System. All primers used for qPCR are available upon request.

### Measurement of P450 enzyme activity

CYP2E1 activity was determined by the rate of oxidation of *p*-nitrophenol (PNP) to *p*-nitrocatechol in the presence of NADPH and O2. The activity was determined using the following equation: PNP activity (in nanomoles per minute per milligram of protein) = D_546_/9.53/0.2/60/7.1 × 10^3^. CYP3A activity was measured using a P450-GloTM CYP3A assay with luciferin-IPA (Promega) according to the instruction. CYP1A2 activity was measured by the rate of 7-ethoxyresorufin O-deethylation (EROD) in the presence of NADPH and O2. EROD was measured by HPLC as previously described in Pastrakuljic A et al^41^.

### LC-MS/MS analysis of PARP1-associating proteins

For LC-MS/MS analysis of PARP1-associating proteins, APAP-treated liver extracts were mixed with anti-PARP1 antibody or IgG antibody, and rotated overnight at 4 °C. The protein-A/G agarose bead was added for another 3 h at 4 °C. After washing with lysis buffer, the immunoprecipitated proteins were subjected to western blot and silver staining. The bands were then subjected to LC-MS/MS analysis. Data were analyzed with Protein Pilot software (AB SCIEX, Framingham, USA).

### Far-Western blot assays

Far-Western blot assays were performed as described previously^20^. Briefly, after SDS-PAGE running, resolving protein samples were transferred to PVDF membranes. Membranes were then incubated overnight at 4 °C with Hyb-75 buffer. Recombinant PARP1 protein (1ug/ml; Trevigen) was incubated with active DNA, NAD^+^, and PARP buffer (from the PARP assay kit) at 37 °C for 1 h to obtain autopoly(ADP-ribosyl)ated PARP1 protein (AP-PARP1). Membranes were washed briefly with Hyb-75 buffer and then incubated with recombinant PARP1 protein, AP-PARP1 protein, recombinant PXR protein (Active Motif, 31144), or recombinant β-actin (Abnova) at 37 °C for 1 h. After washing with Hyb-75 buffer, membranes were incubated with the indicated antibodies at 4 °C overnight. After washing, membranes were incubated with related HRP-conjugated secondary antibodies for 2 h. Chemiluminescence signals were detected by the Image Lab software.

### In vitro poly(ADP-ribosyl)ation assay

Nuclear extracts or recombinant PXR protein were incubated with NAD^+^ and activated DNA in poly(ADP-ribosyl)ation assay buffer (50 mM Tris-HCl [pH8.0], 10 mM MgCl_2_, 1 mM DTT) or with 3AB (10 mM) for 30 min at 37 °C. Poly(ADP-ribosyl)ation of nuclear extracts were then subjected to IP assay.

### Cell cultures

Primary mouse hepatocytes were isolated from C57BL/6 J mice as described previously^20^. Briefly, primary hepatocytes were isolated from mice with perfusion of livers with 0.05% Collagenase Type IV (Sigma-Aldrich). Cells were then plated in 6-well plates in Williams’ E medium (Gibco, CA, USA) supplemented with 10% fetal bovine serum (FBS),1 mM GlutaMAX (Gibco) for 4 h for attachment. After that, cells were cultured in the William’s E medium without serum overnight before APAP treatment. HepG2 cells were purchased from the American Type Culture Collection (ATCC) and were maintained in DMEM supplemented with 10% FBS.

### Luciferase assay

The promoter region of CYP3A11 genes was amplified by PCR and subcloned into pGL3.0-basic (Promega). These constructs were introduced into hepatocytes along with pSV-TK(Promega), which was used to normalize the transfection efficiency. Then cells were treated with PJ34 or APAP as indicated and luciferase activities were measured 24 h later using the Dual-Luciferase Assay System (Promega).

### PXR LBD activity assay

The plasmids expressing the yeast transcription factor GAL4-PXR-LBD (ligand-binding domain), Gal4-PXR-LBD mutant, and a GAL4 reporter (Promega) were constructed and the plasmids were transfected into HepG2 cells. After treatments, cell lysates were harvested to measure luciferase activity. The results were normalized to β-galactosidase activity.

### Chromatin immunoprecipitation (CHIP) and re-CHIP assay

For the CHIP assay, primary hepatocytes were treated according to the procedure. After 48 h, cells were cross-linked with 1% formaldehyde, then harvested, and sonicated to generate DNA fragments of 0.2–1 kb. Lysates were centrifuged. Supernatants were immunoprecipitated with indicated antibodies or an IgG. Finally, DNA was purified using the QIAquick PCR purification kit. Purified DNA was analyzed by real-time PCR with specific primers for CYP3A11 promoter. In re-CHIP assay, chromatin was firstly immunoprecipitated with an anti-PXR antibody, then eluted with 100 μl of elution buffer with 10 mM DTT at 37 °C for 1 h, diluted with dilution buffer and finally re-immunoprecipitated with IgG or an antibody against PARP1, PAR, RNApolII.

### Statistical analysis

Values are shown as the means ± SEM of at least three independent experiments. The statistical significance of differences between two groups was analyzed by Student’s *t* tests. For comparing more than two means, one-way analysis of variance (ANOVA) with the Newman–Keuls post-hoc analysis was employed. Values of *P* < 0.05 were considered statistically significant. All statistical analyses were performed using SPSS software (version 22.0, SPSS Inc).

## Electronic supplementary material

Supplemental material
