# Supplementary material for: Poly(ADP-ribosyl)ated PXR is a critical regulator of acetaminophen-induced hepatotoxicity
Source: Cell Death Dis. 2018 Jul 26;9(8):819. doi: 10.1038/s41419-018-0875-4 (PMC6062506; doi:10.1038/s41419-018-0875-4)
Supplement: Supplementary file 1 — Supplemental material [file 41419_2018_875_MOESM1_ESM.docx]

**Supplementary Data**

**Poly(ADP-ribosyl)ated PXR is a critical regulator of acetaminophen-induced hepatotoxicity**

Cheng Wang^1,2,*^, Wenjing Xu^1,2,*^, Yanqing Zhang^1^, Dan Huang^1,2,^ , Kai Huang^1,2, #^

1. Clinic Center of Human Gene Research, Union Hospital, Tongji Medical College, Huazhong University of Science and Technology, Wuhan, China.

2. Department of Cardiology, Union Hospital, Tongji Medical College, Huazhong University of Science and Technology, Wuhan, China.

* These authors contributed equally to this work

^#^Correspondence to: Kai Huang, Clinic Center of Human Gene Research, Union Hospital, Tongji Medical College, Huazhong University of Science and Technology, 1277 Jiefang Ave, Wuhan 430000, China. E‐mail: huangkaiunion@gmail.com

**Short Title:** Poly(ADP-ribosyl)ated PXR in hepatotoxicity

**Key Words:**

Acetaminophen; poly(ADP-ribose) polymerase 1; PXR; Liver injury; transcriptional regulation

Supplementary figures


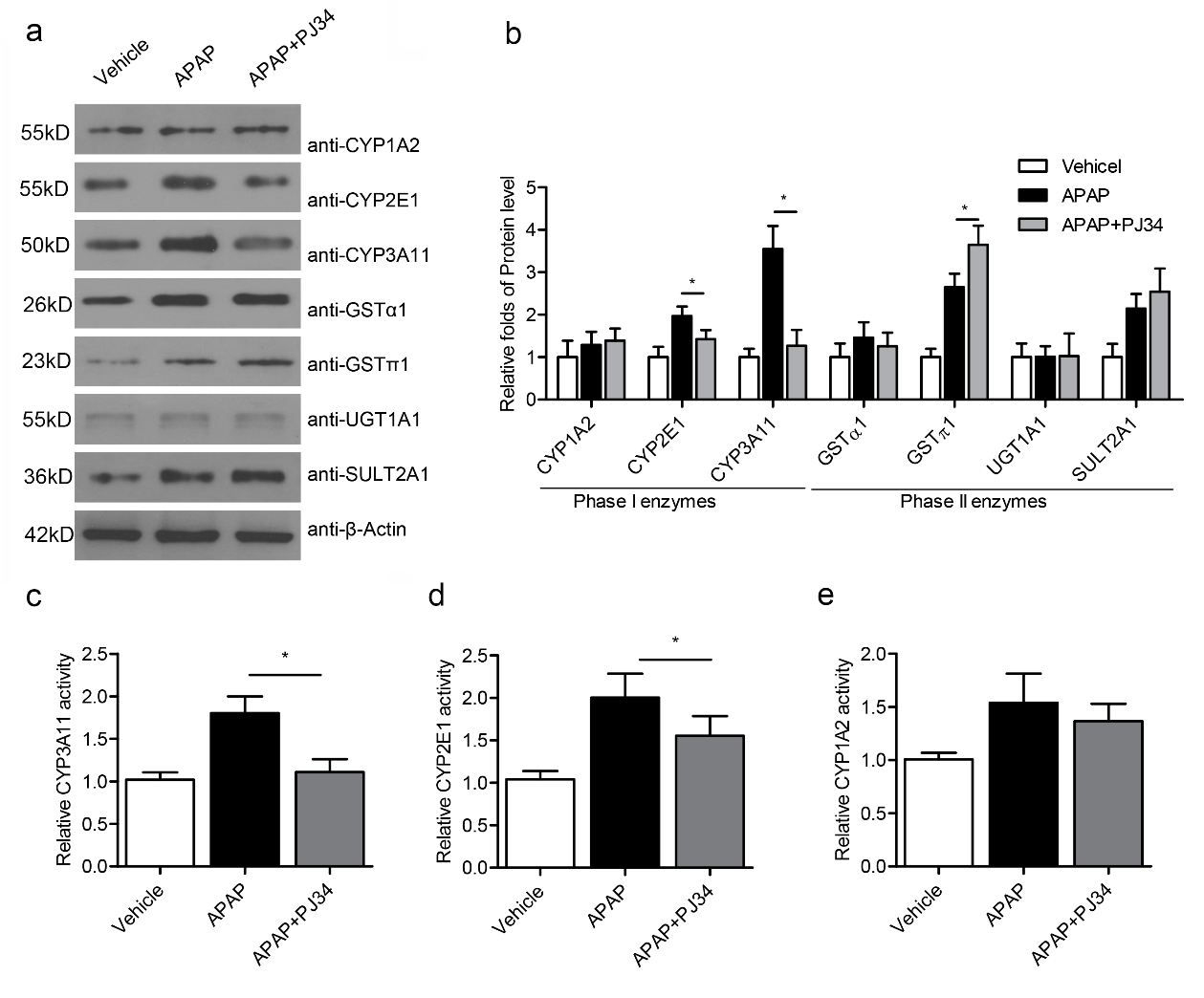


**Supplementary Fig.1** PARP inhibitor PJ34 regulates APAP metabolism enzymes expression and activity. C57BL/6J mice were injected intraperitoneally with PJ34, and then administered with 300 mg/kg APAP injection. Livers were collected at 24 hours after APAP injection. (**a**) The proteins levels of indicated genes were detected by Western blot and (**b**) the relative quantification. (**c**) The activity of CYP3A11 in livers by luciferin-IPA. (**d**) The activity of CYP2E1 in livers by PNP oxidation. (**e**) The activity of CYP1A2 in livers by EROD.


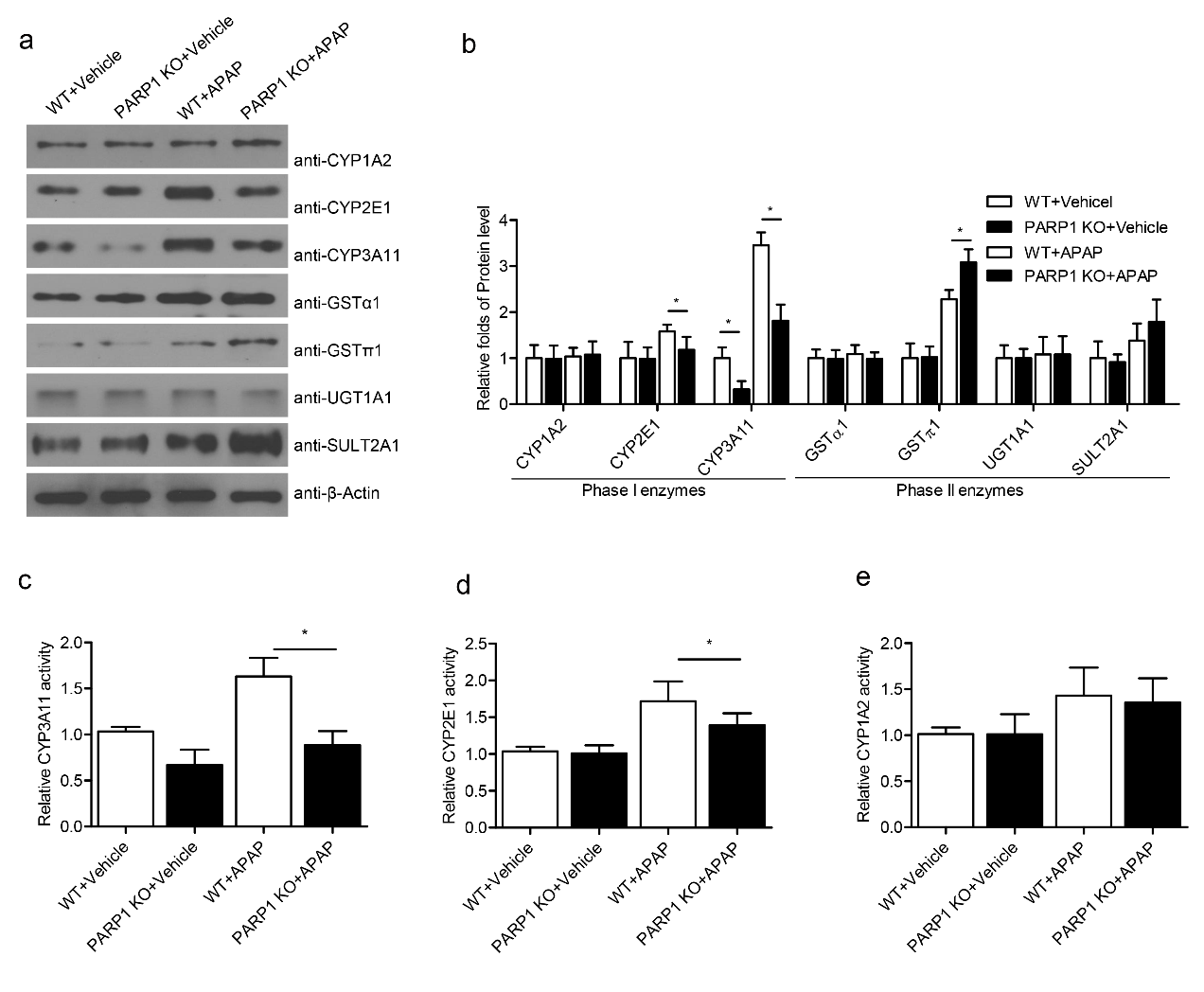


**Supplementary Fig.2** PARP1 depletion suppresses APAP toxic metabolism enzymes expression and activity. WT and PARP1^-/-^ mice were injected intraperitoneally with APAP (300 mg/kg). liver samples were collected at 24 hours later. (**a**) The proteins levels of indicated genes were detected by Western blot and (**b**) the relative quantification. (**c**) The activity of CYP3A11 in livers by luciferin-IPA. (**d**) The activity of CYP2E1 in livers by PNP oxidation. (**e**) The activity of CYP1A2 in livers by EROD.


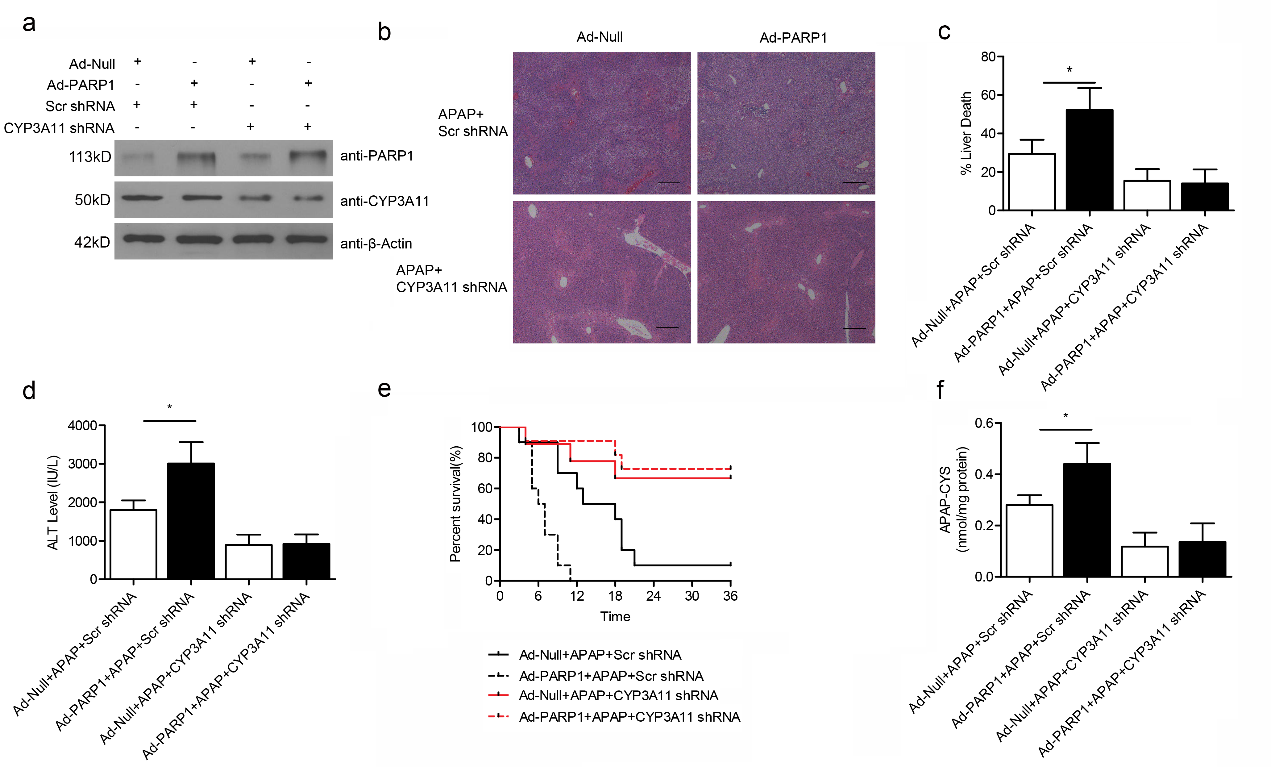


**Supplementary Fig.3** CYP3A11 deficiency relieves the pro-effects of PARP1 in APAP metabolism and liver toxicity. C57BL/6J mice were pre-delivered Ad-Null or Ad-PARP1, together with Ad-Scr shRNA or Ad-CYP3A11 shRNA adenovirus through tail vein, and then exposed to APAP treatment (300 mg/kg). Blood and tissue were collected at indicated times. (**a**) Representative western blot analysis of hepatic PARP1 and CYP3A11 expressions. (**b**) Representative H&E staining of liver sections from indicated groups. (**c**) Quantification of liver death area. (**d**) Serum levels of ALT activity. (**e**) Mice were treated with a lethal dose of APAP (1 g/kg). Survival was followed for 36 h post administration from indicated groups. (**f**) Hepatic APAP-cysteine levels at 6 hours after APAP treatment from indicated groups. N=10-12 for each group. **P*<0.01 vs. APAP.
